# Analysis of the Relationship of Glycated Hemoglobin with Subclinical Atherosclerosis and Arterial Stiffness in Non-Diabetic Patients: A Retrospective Study

**DOI:** 10.3390/jcm15103627

**Published:** 2026-05-09

**Authors:** Grzegorz K. Jakubiak, Natalia Pawlas, Dominika Blachut, Artur Chwalba, Andrzej Tomasik, Agata Stanek, Grzegorz Cieślar

**Affiliations:** 1 Department of Pharmacology, Faculty of Medical Sciences in Zabrze, Medical University of Silesia in Katowice, Jordana 38 St., 41-808 Zabrze, Poland; n-pawlas@wp.pl (N.P.); artur.adam.chwalba@gmail.com (A.C.); 2 2nd Department of Cardiology, Faculty of Medical Sciences in Zabrze, Medical University of Silesia in Katowice, Skłodowskiej-Curie 10 St., 41-800 Zabrze, Poland; dominikadyrcz@gmail.com (D.B.); tomasik@poczta.onet.pl (A.T.); 3 Department of Internal Medicine, Metabolic Diseases and Angiology, Faculty of Health Sciences in Katowice, Medical University of Silesia in Katowice, Ziolowa 45/47 St., 40-635 Katowice, Poland; astanek@o2.pl; 4 Department of Internal Medicine, Angiology and Physical Medicine, Faculty of Medical Sciences in Zabrze, Medical University of Silesia in Katowice, Batorego 15 St., 41-902 Bytom, Poland; cieslar1@tlen.pl

**Keywords:** glycated hemoglobin, non-diabetic patients, intima–media thickness, ankle–brachial index, toe–brachial index, pulse wave velocity, arterial stiffness, atherosclerosis

## Abstract

**Background:** Cardiometabolic diseases present a major challenge to contemporary public health. Diabetes mellitus (DM) is widely recognized as a strong cardiovascular risk factor. However, the utility of glycated hemoglobin percentage (HbA1c) for assessing cardiovascular health in individuals without DM remains uncertain. This study examines the association between HbA1c levels and both the presence and severity of subclinical atherosclerosis and arterial stiffness in non-diabetic individuals. **Methods:** A retrospective analysis was conducted on the data from 59 patients (72.88% female; mean age: 54.82 ± 17.34 years) who exhibited no signs of acute illness or exacerbation of chronic diseases. All patients were hospitalized in the Department of Internal Medicine, Angiology and Physical Medicine at the Medical University of Silesia in Katowice, Poland, between June 2022 and May 2024. HbA1c level determination, central blood pressure measurement with pulse wave analysis (PWA), carotid–femoral pulse wave velocity (cfPWV) measurement, and Doppler ultrasound of the carotid arteries and lower extremity arteries measuring the intima–media thickness (IMT) in the common carotid arteries (cIMT), common femoral arteries (cfIMT), and superficial femoral arteries (sfIMT) were performed. Spearman’s rank correlation test was applied for statistical analysis. Subsequently, a multivariate analysis model was constructed, adjusting for age, sex, body mass index (BMI), and smoking. **Results:** Among the assessed parameters, the strongest positive correlations were found between HbA1c and parameters such as cIMT (R = 0.532; *p* < 0.001), cfIMT (R = 0.63; *p* < 0.001), sfIMT (R = 0.539; *p* < 0.001), and cfPWV (R = 0.504; *p* < 0.001). In the multivariate analysis model, a significant relationship was found only between HbA1c and augmentation index normalized to a heart rate of 75 per minute (AIx75) (*β* = −0.286; 95% CI: −0.566, −0.006; *p* = 0.045). **Conclusions**: In summary, although HbA1c correlates with some parameters related to arterial stiffness and subclinical atherosclerosis in non-diabetic patients, most observed relationships are explained by confounding variables.

## 1. Introduction

Cardiovascular diseases (CVDs) constitute a significant global public health challenge [1]. Despite substantial progress in pharmacological and invasive therapies, CVDs remain a primary cause of morbidity and mortality around the world [2]. Effective prevention relies on the precise identification and management of modifiable cardiovascular risk factors [3].

Measurement of glycated hemoglobin percentage (HbA1c) is a fundamental diagnostic tool for assessing average serum glucose concentrations measured over the past three months [4]. The HbA1c test is used both to diagnose diabetes mellitus (DM) and to evaluate glycemic control in patients undergoing treatment for established DM [5]. Hyperglycemia at levels meeting the diagnostic criteria for DM is undoubtedly one of the most potent cardiovascular risk factors [6]. Recently, the possibility of using HbA1c to assess cardiovascular risk in individuals without DM has attracted growing interest [7]. The feasibility of this approach depends on whether subclinical glycemic fluctuations that do not meet the threshold for DM diagnosis but result in elevation in HbA1c values are associated with clinically significant cardiovascular risk and the progression of subclinical cardiovascular dysfunction. We recently published a paper examining the associations between HbA1c and selected parameters obtained by transthoracic echocardiography in patients without DM. We then showed that the observed relationships in univariate analysis could be fully explained by the influence of confounding variables [8]. In this study, using data from the same study population, we analyzed the relationship between HbA1c and both arterial stiffness (assessed by pulse wave velocity and pulse wave analysis parameters) and subclinical atherosclerosis (assessed by ankle–brachial index, toe–brachial index, and intima–media thickness in different vascular beds).

The purpose of this study was to assess the relationship between HbA1c and selected subclinical atherosclerosis and arterial stiffness parameters in non-diabetic patients. We analyzed intima–media thickness in different vascular beds (common carotid artery, common femoral artery, and superficial femoral artery), as well as ankle–brachial index (ABI), toe–brachial index (TBI), pulse wave velocity (PWV), and selected parameters of pulse wave analysis (PWA).

## 2. Materials and Methods

### 2.1. Study Population

This study involved a retrospective analysis of data from patients admitted to the Department of Internal Medicine, Angiology and Physical Medicine at the Medical University of Silesia in Katowice (Poland) between June 2022 and May 2024.

This study included non-diabetic individuals hospitalized for planned diagnostics, without any signs of acute illness or exacerbation of chronic diseases in the month preceding enrollment, who underwent HbA1c measurement and cardiovascular system assessments during hospitalization. The assessment of carbohydrate metabolism and the classification of patients as non-diabetic were based on data from medical history, currently used medications, current fasting plasma glucose levels, and current HbA1c values. The detailed inclusion and exclusion criteria are presented in Table 1. The inclusion and exclusion criteria are the same as those presented in the previous publication, which was based on the same population [8].

### 2.2. Laboratory Tests

Laboratory tests were performed at the Laboratory of Specialist Hospital No. 2 in Bytom. Blood samples were collected in the morning following a fasting period of approximately 12 to 14 h.

HbA1c levels were measured with a turbidimetric inhibition immunoassay with the Tina-quant Hemoglobin A1c Gen.3 kit (Roche Diagnostics GmbH, Mannheim, Germany).

### 2.3. Assessment of the Cardiovascular System

Arterial Doppler ultrasound examinations were conducted according to the current guidelines [9,10,11] using an RS80 EVO device (Samsung Medison Co., Ltd., Gangwon-do, Republic of Korea) equipped with a linear probe LA4-18B or LA3-12A (for the examination of carotid arteries or lower extremity arteries, respectively). Importantly, all Doppler examinations were performed by the same physician, who was experienced in performing them. Intima–media thickness (IMT) was measured manually within the common carotid artery one centimeter below the bifurcation (cIMT), within the common femoral artery one centimeter above the bifurcation (cfIMT), and within the superficial femoral artery one centimeter below the bifurcation (sfIMT). Each measurement was repeated three times, and the arithmetic mean was recorded in the patient’s medical history. For each anatomical location, the arithmetic mean of the right- and left-sided values was used for the final analysis.

The ABI was measured bilaterally with a Dopplex Ability Automatic ABI System (Huntleigh Healthcare Ltd., Cardiff, UK). The TBI was measured bilaterally using a Dopplex DMX Digital Doppler (Huntleigh Healthcare Ltd., Cardiff, UK). For the final analysis, the mean value of the ABI and the mean value of the TBI from both sides were calculated.

Central blood pressure, PWA parameters, and carotid–femoral PWV (cfPWV) were measured with the Sphygmocor XCEL device (AtCorMedical, Sydney, Australia).

All procedures were performed in thermally comfortable conditions after patients had rested in the supine position for at least 10 min.

### 2.4. Statistical Analysis

Counts and percentages were used to present qualitative variables. The quantitative variable presentation depended on the empirical distribution and its similarity to a normal distribution. The mean and standard deviation were used when distributions approximated normality or the median and both first and third quartiles when significant deviations from normality were detected. Normality was evaluated using the Shapiro–Wilk and Kolmogorov–Smirnov tests, as well as the visual inspection of histograms. Associations between quantitative variables were assessed using the Spearman rank correlation test. A *p*-value lower than 0.05 was considered to be statistically significant.

After conducting univariate correlation analyses, a multivariate model was developed to clarify the association between HbA1c and parameters related to subclinical atherosclerosis and arterial stiffness, with adjustment for potential confounders. The generalized linear model (GLM) was applied. Confounding variables were selected based on the results of univariate analyses, including correlation analyses. Finally, the model was adjusted for age, sex, body mass index (BMI), and smoking status. Variables that did not follow a normal distribution were subjected to logarithmic transformation. For some variables, the model could not be built due to the distribution not being in line with the normal distribution and the failure of the logarithmic transformation attempt.

Missing data were addressed using a complete-case approach (listwise deletion). Taking into consideration the low proportion of missing values, this method was unlikely to introduce significant bias or compromise the robustness of the results.

Since this study was exploratory and had a limited sample size, no formal correction for multiple testing was applied. Applying such corrections in small datasets can increase the risk of type II errors and may obscure potentially meaningful associations. Therefore, the results should be interpreted with caution.

Statistical analyses were performed using Statistica (data analysis software system), version 13 (TIBCO Software Inc., Palo Alto, CA, USA, 2017).

## 3. Results

### 3.1. Basic Characteristics of the Study Population

A total of 59 individuals (72.88% female) were included in the final analysis. Their mean age was 54.82 ± 17.34 years, and their mean BMI was 25.81 ± 4.38 kg/m^2^. Their median fasting plasma glucose level was 91.3 mg/dL (85.8; 96.0), and their median HbA1c was 5.46% (5.29; 5.62). Their median non-high-density lipoprotein cholesterol (non-HDL-C) was 136.9 mg/dL (110.8; 160.0). Their median triglyceride (TG) serum concentration was 100.0 mg/dL (70.0; 134.0). Within the study group, 30 individuals (50.85%) received treatment for hypertension. Four participants were diagnosed with atrial fibrillation. Additionally, 31 individuals (52.54%) were diagnosed with atherosclerosis based on the presence of atherosclerotic plaques identified in imaging studies. However, none of the participants had a history of cardiovascular events or revascularization in any vascular bed. Creatinine serum concentration ranged from 0.48 mg/dL to 1.2 mg/dL in the study population. None of the study participants were taking any medications that could significantly affect carbohydrate metabolism.

Comprehensive details regarding the baseline characteristics of the study population are available in a previous publication [8].

### 3.2. Descriptive Statistics of Parameters Related to Cardiovascular Assessment

Most individuals in the study population exhibited parameter values within the normal range. An ABI below 0.9 was observed in three individuals (5.08%), while four individuals (6.78%) had an ABI between 0.9 and 1.0. No participant demonstrated an ABI above 1.3. A higher proportion of patients (32.2%) had a TBI below 0.7. However, noteworthily, a TBI of <0.6 was found in 11.86% cases, while the TBI was in the range of 0.6–0.7 in 20.34% cases. Additionally, 10 individuals (16.95%) exhibited a cfPWV greater than 10 m/s, and 16 individuals (27.12%) had a cIMT exceeding 0.9 mm.

Table 2 provides a detailed summary of the cardiovascular parameters assessed in the entire study population.

### 3.3. Correlation of Glycated Hemoglobin with Cardiovascular Parameters

Within the study population, a significant correlation between HbA1c and IMT was observed across all analyzed vascular beds. This association persisted when IMT values were assessed separately for the right and left sides, as well as for minimum, maximum, and mean measurements. The strongest correlation was identified for cfIMT, whereas slightly weaker correlations were noted for sfIMT and cIMT.

No significant correlation was identified between HbA1c and either ABI or TBI. However, significant correlations were observed between HbA1c and both cfPWV and cPP, but not with cSBP or cDBP. Regarding PWA parameters, significant correlations were found between HbA1c and augmentation pressure (AP), backward pressure (Pb), and forward pressure (Pf), while no significant associations were observed for the remaining parameters.

The complete results of the correlation analysis between HbA1c and subclinical atherosclerosis and arterial stiffness parameters are provided in Table 3.

### 3.4. Results of the Multivariate Analysis

The created model, adjusted for age, sex, BMI, and smoking, was found to be significant for the following parameters: cIMT, log-transformed cfPWV [log(cfPWV)], cSBP, cPP, cMAP, MAP systole, log-transformed PTI systole [log(PTI systole)], log-transformed AP [log(AP)], AIx, AIx75, ESP, Pb, log-transformed Pf [log(Pf)], log-transformed PPA [log(PPA)], and RM. The complete results are presented in Table 4. For the remaining parameters related to the cardiovascular system status, which are not presented in Table 4, it was not possible to construct a model because, on the one hand, the empirical distributions of these variables differed significantly from the normal distribution, and, on the other hand, these variables failed to be log-transformed.

In the further analysis, only the influence of HbA1c values on the results was examined. A significant influence of HbA1c on the results was found only for AIx75. For other variables, the relationships between HbA1c and parameters related to cardiovascular dysfunction were fully explained by confounding factors. The complete results are presented in Table 5.

## 4. Discussion

Subclinical atherosclerosis represents a significant public health concern. A study involving children examined cardiometabolic risk factors and found that early metabolic disturbances, including elevated glucose levels, insulin resistance, and abnormal lipid profiles, are associated with the early markers of vascular dysfunction. The presence of these adverse factors at a young age predisposes individuals to the development of an unfavorable vascular phenotype in later life. The early identification of metabolic risk factors may facilitate timely intervention and limit the progression of arterial stiffness and cardiovascular complications [12].

This retrospective study included patients without a prior diagnosis of DM and evaluated the association between HbA1c levels and subclinical atherosclerosis and arterial stiffness parameters. The study group comprised individuals in whom atherosclerosis was detected during assessment. An ABI below 0.9 was uncommon, occurring in fewer than 5% of the participants, and an ABI above 1.3 was not observed, indicating a low prevalence of peripheral artery disease. In contrast, a reduced TBI (below 0.7) was observed in approximately 30% of the participants, suggesting changes in distal vessels. Increased cfPWV (above 10 m/s), indicative of large-artery stiffness, was observed in approximately 17% of the participants, while elevated cIMT (above 0.9 mm) was present in more than 25% of the participants. These findings demonstrate that, despite the absence of diagnosed DM, the study population exhibited features of subclinical vascular impairment and arterial wall remodeling. In univariable analyses, HbA1c was positively associated with the markers of subclinical atherosclerosis and arterial stiffness. Strong correlations were observed between HbA1c and IMT, cfPWV, and cPP. HbA1c showed significant correlations with IMT across all analyzed vascular beds (cIMT, cfIMT, sfIMT), with no laterality effects. The most pronounced association was identified in the common femoral artery. However, it should be emphasized that most observed relationships did not remain significant after considering the influence of confounding factors (age, sex, BMI, and smoking). The relationship remained significant only for AIx75.

Previous studies have reported associations between chronically elevated glucose concentrations and subclinical atherosclerosis markers, as well as the severity of arterial wall remodeling [13]. Increased IMT is a well-established marker of subclinical atherosclerosis and reflects cardiovascular risk, while aortic stiffness—assessed indirectly using cfPWV—has well-recognized prognostic value [14]. Prospective data and meta-analyses indicate that cfPWV is useful in cardiovascular risk assessment [15,16,17,18]. In a Chinese population study, higher baseline HbA1c was associated with a significantly greater risk of arterial stiffness progression during follow-up, independent of traditional cardiovascular risk factors. The authors suggested that chronic exposure even to moderate hyperglycemia may promote adverse vascular remodeling via enhanced non-enzymatic protein glycation, generation of advanced glycation end-products (AGEs), and alterations in extracellular matrix structure, leading to sustained increases in vascular stiffness [19].

In patients with type 1 DM, higher HbA1c variability was found to be associated with increased arterial stiffness [20], suggesting that a single HbA1c measurement may be insufficient and that not only chronic hyperglycemia but also short-term glucose fluctuations may affect the vascular phenotype. HbA1c may also represent a relevant component of metabolic dysregulation, including insulin resistance. Data from non-diabetic populations indicate that aortic dysfunction may be detectable at the insulin resistance stage despite a normal oral glucose tolerance test (OGTT). Stakos et al. reported that, in individuals without DM and with normal OGTT results, indices of ascending aortic function such as the stiffness index were associated with the severity of insulin resistance; moreover, during the one-year follow-up, deterioration was observed in individuals with more pronounced homeostasis model of assessment of insulin resistance (HOMA-IR), suggesting that metabolic abnormalities influencing vascular remodeling may precede overt dysglycemia [21]. Similar studies suggest that insulin resistance may be an important common denominator linking HbA1c and vascular stiffness [22,23,24]. Chen et al. demonstrated that a higher Stress Hyperglycemia Ratio (SHR) was significantly associated with greater arterial stiffness, independent of mean glycemia and other cardiovascular risk factors. These data support HbA1c variability control as a predictor of stiffness progression [25,26,27,28,29,30,31,32]. Overall, despite the absence of DM, patients may be exposed to premature atherosclerosis related to higher mean glycemia.

In the study by Cederqvist et al., arterial stiffness increased with the severity of dysglycemia and was significantly associated with the presence of subclinical coronary atherosclerosis, independent of traditional cardiovascular risk factors [33]. Moreover, indices such as the hemoglobin glycation index were associated with higher cardio-ankle vascular index (CAVI), independent of hyperglycemia, suggesting that individual susceptibility to glycation may represent a relevant mechanism of vascular risk [34]. In our study, HbA1c was measured once; the OGTT was not performed, and insulin resistance was not assessed, probably resulting in the limited characterization of the study population. In a CAVI-based study in patients with type 2 DM, no correlation between HbA1c and CAVI was observed, whereas systolic blood pressure was an independent predictor [35]. The coexistence of DM and hypertension significantly increased the risk of abnormal CAVI [34,36,37]. This finding underscores that, in multivariable models, hemodynamic factors may dominate over markers of dysglycemia, which may also be consistent with our results showing the reduced independence of HbA1c after multivariable adjustment. Similarly, Cameron et al. reported differences in arterial aging in DM compared with non-diabetic individuals, suggesting the accelerated stiffening of elastic arteries at a younger age and an altered age–stiffness relationship later in life, which may affect the interpretation of linear associations between HbA1c and cfPWV depending on disease duration and blood pressure control [38]. Higher aortic stiffness is associated with an increased long-term risk of adverse health outcomes, independent of traditional cardiovascular risk factors. In multivariable analyses, these associations persisted after accounting for blood pressure and metabolic parameters [39].

Aortic stiffness leads to increased PWV and an earlier return of the reflected wave. Increased cfPWV promotes microvascular damage, while earlier wave reflection increases afterload and contributes to left ventricular dysfunction. Consequently, aortic stiffness increases cardiovascular risk [40,41,42,43]. In correlation analyses, HbA1c showed a significant positive association with cfPWV and central pulse pressure (cPP), but not with cSBP or cDBP. This suggests that HbA1c was mainly related to parameters reflecting pulsatile blood pressure variability rather than the steady component of blood pressure. cfPWV is an index of vascular aging and arterial stiffness. In our analysis, higher HbA1c was present in approximately one-sixth of participants with increased arterial stiffness, which may indicate that the association between HbA1c and aortic stiffness is secondary to established vascular aging determinants such as age and obesity. Prior studies have largely focused on individuals with type 2 DM or overt hyperglycemia, consistently demonstrating associations between glycemic control metrics and cfPWV. In the ELSA-Brasil study, individuals with DM exhibited higher cfPWV, and cfPWV increased progressively with the fulfillment of laboratory criteria used to diagnose DM (fasting glucose, HbA1c, and 2 h post-load glucose), suggesting that vascular stiffness may reflect dysglycemia [44]. Similar findings were reported in the Malmö Offspring Study assessing the relationship between glycemic markers and cfPWV. cfPWV values were markedly higher in the older generation, and hyperglycemia likely amplified the vascular aging process. Notably, differences in cfPWV between individuals with and without DM were observed in both generations [45].

Studies have shown that higher cfPWV is associated with an increased risk of incident DM during follow-up, supporting the hypothesis that hemodynamic alterations and arterial stiffness may contribute to the pathogenesis of metabolic dysfunction through microvascular injury in high-flow organs (heart, kidneys) and exacerbation of insulin resistance [15,46,47]. Additionally, evidence suggests that increased arterial stiffness—particularly when combined with hypertension—raises the risk of incident DM, further emphasizing the role of blood pressure and arterial stiffness as components of a feedback loop between the cardiovascular system and metabolic dysregulation [46,48,49,50,51,52,53]. Greve et al. showed that the estimated cfPWV has predictive value comparable to the measured cfPWV and improves risk stratification. However, the estimated cfPWV depends on age and blood pressure, which may inflate apparent associations with other variables in cross-sectional analyses [54]. It is also worth noting another stiffness-related metric such as the inter-limb difference in digital volume pulse (DVP). The authors reported that patients with DM had greater DVP differences, correlating with the extent of arteriosclerosis and arterial stiffness; such DVP differences may reflect early, subtle vascular changes related to DM that may not be captured by standard assessment methods [55].

In multivariable analysis, the only parameter that remained significantly associated with HbA1c was AIx75, with an inverse relationship. AIx75 is a waveform-derived parameter influenced by multiple hemodynamic determinants, including the timing and amplitude of wave reflection, peripheral vascular tone, and left ventricular ejection characteristics. AIx75 reflects the partially distinct aspects of hemodynamics and is less stable than cfPWV. In this study, the inverse association between AIx75 and HbA1c may correspond to a hemodynamic phenotype related to microcirculation rather than being strictly aortic. It should also be recognized that segmental studies demonstrate heterogeneity of stiffness across different vascular beds (elastic vs. muscular) and support the concept of accelerated “vascular aging” in type 2 DM, further suggesting that waveform indices and cfPWV may not change in parallel. Therefore, the AIx75 finding should be interpreted as exploratory and requires confirmation in larger prospective cohorts [13,38,42].

The study population included individuals with a normal ABI but a reduced TBI, a subgroup that warrants further discussion. This phenomenon has been documented in the literature. For example, a large study conducted in Denmark reported that 20.5% of the participants exhibited this characteristic. According to the authors of this study, the effect of reduced TBI on cardiovascular risk is similar to the effect of reduced ABI [56]. In another study, conducted only among patients with diabetes, it was shown that age, diabetic duration, and BMI are risk factors for a reduced TBI with a normal ABI value [57]. The range of reference values for the TBI remains controversial and has varied considerably among researchers [58].

### Limitations of the Study

The key limitations of this study include its cross-sectional design, which only allows us to determine the existence of certain dependencies, without the possibility of drawing conclusions about the cause-and-effect relationship, and the small sample size, limiting statistical power and increasing susceptibility to multivariable model instability. Considering the size of the population and the number of parameters analyzed, the risk of error increases. Conducting the study with a larger population would enable the development of additional models that could consider more potential confounding variables and their interactions. In the current dataset, these possibilities were limited, which means we should be cautious when interpreting the results. The single-center design remains a significant limitation.

Due to the retrospective nature of the study, there are some missing data in the collected dataset. Since the presented results are a continuation of a previous study [8], the inclusion criteria included echocardiography during hospitalization. However, because the Department where the data were collected also assessed other cardiovascular parameters, the missing data were relatively small.

HbA1c was assessed once, without evaluating its temporal variability, which restricts a more comprehensive characterization of dysglycemia. Furthermore, a significant limitation of this study is the lack of direct assessment of insulin resistance, which would be a valuable addition to the analysis conducted.

Moreover, different methods and measurement segments for stiffness assessment (cfPWV vs. waveform indices) are not equivalent, and their interpretation requires the consideration of hemodynamic context, measurement reproducibility, and potential effects of therapy and comorbidities. A significant limitation of our study, in terms of the methodology for assessing the cardiovascular system, was the lack of assessment of endothelial function, which could be significantly influenced by subclinical glycemia fluctuations [59].

Additionally, the lack of long-term clinical endpoints precludes the assessment of the prognostic significance of the observed associations. Our results—obtained in a population without overt DM—should therefore be interpreted as correlational relationships that are largely attenuated after adjusting for age and metabolic profile.

Future studies should include larger population-based cohorts with prospective follow-up and multicenter recruitment, repeated HbA1c measurements, and expanded vascular phenotyping to determine whether glycemic indices add value beyond the classical risk factors and reference measures of arterial stiffness.

## 5. Conclusions

Our study results show that, although the HbA1c value correlates with some parameters related to arterial stiffness and subclinical atherosclerosis in patients without DM, multivariate analyses indicate that, in almost every case (except AIx75), the observed relationships are fully explained by confounding variables. Further studies are needed, especially multicenter, prospective studies on larger groups of patients, which would allow the better verification of the relationship between HbA1c and subclinical atherosclerosis and arterial stiffness parameters in non-diabetic patients, as well as the assessment of their clinical significance.

## Figures and Tables

**Table 1:** Inclusion and exclusion criteria.

Inclusion Criteria	Exclusion Criteria
(1) Age at least 18 years; (2) Hospitalization for planned internal medicine diagnostics; (3) Echocardiography performed during hospitalization; (4) HbA1c test performed during hospitalization.	(1) Diagnosed diabetes or prediabetes; (2) Any acute illness or exacerbation of chronic disease within the month preceding their admission to the hospital; (3) Tachyarrhythmia; (4) Features of decompensated heart failure; (5) Respiratory failure; (6) Anemia; (7) Infection; (8) Diagnosed malignant neoplasm; (9) Chronic kidney disease.

**Table 2:** Parameters related to the subclinical atherosclerosis and arterial stiffness in the entire study population.

Parameter	Mean (SD)/Median (Q1; Q3)	Minimal Value	Maximal Value
cIMT [mm]	0.735 (0.615; 0.965)	0.45	1.29
cfIMT [mm]	0.715 (0.593; 1.3)	0.38	5.4
sfIMT [mm]	0.558 (0.493; 0.678)	0.3	3.4
ABI	1.16 (1.08; 1.21)	0.62	1.29
TBI	0.796 (0.186)	0.33	1.19
cfPWV [m/s]	7.3 (6.3; 9.2)	4.0	23.2
cSBP [mmHg]	117.0 (107.0; 132.0)	91.0	173.0
cDBP [mmHg]	78.0 (73.0; 84.0)	59.0	107.0
cPP [mmHg]	37.0 (31.0; 46.0)	19.0	82.0
cMAP [mmHg]	94.0 (86.0; 101.0)	74.0	140.0
MAP systole [mmHg]	105.0 (97.0; 118.0)	87.0	155.0
MAP diastole [mmHg]	87.0 (81.0; 95.0)	65.0	128.0
PTI systole [mmHg × s/min]	2095.0 (1915.0; 2420.0)	1479.0	4083.0
PTI diastole [mmHg × s/min]	3469.0 (3204.0; 3760.0)	2365.0	4497.0
AP [mmHg]	9.0 (7.0; 17.0)	−3.0	32.0
AIx [%]	27.41 (14.52)	−16.0	62.0
AIx75	24.69 (14.64)	−16.0	67.0
ESP [mmHg]	108.0 (100.0; 120.0)	87.0	161.0
HR duration [ms]	888.9 (145.44)	547.0	1258.0
ED [ms]	299.24 (27.32)	245.0	372.0
SEVR	161.61 (30.42)	96.0	246.0
Pf [mmHg]	27.0 (23.0; 32.0)	18.0	56.0
Pb [mmHg]	16.0 (14.0; 21.0)	8.0	32.0
PPA [%]	132.0 (124.0; 139.0)	109.0	171.0
RM [%]	61.88 (10.89)	38.0	86.0

Abbreviations: cIMT—carotid intima–media thickness; cfIMT—common femoral intima–media thickness; sfIMT—superficial femoral intima–media thickness; ABI—ankle–brachial index; TBI—toe–brachial index; cfPWV—carotid–femoral pulse wave velocity; cSBP—central systolic blood pressure; cDBP—central diastolic blood pressure; cPP—central pulse pressure; cMAP—central mean arterial pressure; MAP systole—mean arterial pressure during systole; MAP diastole—mean arterial pressure during diastole; PTI systole—pressure–time integral during systole; PTI diastole—pressure–time integral during diastole; AP—augmentation pressure; AIx—augmentation index; AIx75—augmentation index normalized to a heart rate of 75 beats per minute; ESP—end-systolic pressure; HR duration—heart rate duration; ED—ejection duration; SEVR—subendocardial viability ratio; Pb—backward pressure wave; Pf—forward pressure wave; PPA—pulse pressure amplification; RM—reflection magnitude.

**Table 3:** Correlations between glycated hemoglobin percentage and subclinical atherosclerosis and arterial stiffness parameters.

Parameter	N	R	*p*
cIMT	59	0.532	<0.001
cfIMT	56	0.63	<0.001
sfIMT	56	0.539	<0.001
ABI	59	0.001	0.996
TBI	59	−0.017	0.899
cfPWV	58	0.504	<0.001
cSBP	59	0.211	0.107
cDBP	59	−0.106	0.425
cPP	59	0.407	0.001
cMAP	59	0.034	0.801
MAP systole	59	0.158	0.231
MAP diastole	59	−0.058	0.662
PTI systole	59	0.009	0.948
PTI diastole	59	−0.014	0.917
AP	59	0.265	0.042
AIx	59	−0.091	0.494
AIx75	59	−0.027	0.837
ESP	59	0.145	0.272
HR duration	59	0.24	0.068
ED	59	0.243	0.064
SEVR	59	−0.025	0.853
Pb	59	0.367	0.004
Pf	59	0.288	0.027
PPA [%]	59	−0.202	0.124
RM	59	0.06	0.651

Abbreviations: cIMT—carotid intima–media thickness; cfIMT—common femoral intima–media thickness; sfIMT—superficial femoral intima–media thickness; ABI—ankle–brachial index; TBI—toe–brachial index; cfPWV—carotid–femoral pulse wave velocity; cSBP—central systolic blood pressure; cDBP—central diastolic blood pressure; cPP—central pulse pressure; cMAP—central mean arterial pressure; MAP systole—mean arterial pressure during systole; MAP diastole—mean arterial pressure during diastole; PTI systole—pressure–time integral during systole; PTI diastole—pressure–time integral during diastole; AP—augmentation pressure; AIx—augmentation index; AIx75—augmentation index normalized to a heart rate of 75 beats per minute; ESP—end-systolic pressure; HR duration—heart rate duration; ED—ejection duration; SEVR—subendocardial viability ratio; Pb—backward pressure wave; Pf—forward pressure wave; PPA—pulse pressure amplification; RM—reflection magnitude.

**Table 4:** Results of the multivariate analysis model significance test for explaining parameters related to cardiovascular dysfunction. The model was adjusted for age, sex, body mass index (BMI), and smoking.

Parameter	Multiple R^2^	*p*
cIMT	0.658	<0.001
log(ABI)	0.077	0.647
log(cfPWV)	0.546	<0.001
cSBP	0.372	<0.001
cDBP	0.15	0.199
cPP	0.536	<0.001
cMAP	0.216	0.045
MAP systole	0.312	0.003
MAP diastole	0.183	0.097
log(PTI systole)	0.229	0.032
PTI diastole	0.138	0.246
log(AP)	0.508	<0.001
AIx	0.38	<0.001
AIx75	0.359	<0.001
ESP	0.325	0.002
HR duration	0.105	0.44
ED	0.146	0.215
SEVR	0.157	0.17
Pb	0.52	<0.001
log(Pf)	0.469	<0.001
log(PPA)	0.38	<0.001
RM	0.363	<0.001

Abbreviations: cIMT—carotid intima–media thickness; ABI—ankle–brachial index; cfPWV—carotid–femoral pulse wave velocity; cSBP—central systolic blood pressure; cDBP—central diastolic blood pressure; cPP—central pulse pressure; cMAP—central mean arterial pressure; MAP systole—mean arterial pressure during systole; MAP diastole—mean arterial pressure during diastole; PTI systole—pressure–time integral during systole; PTI diastole—pressure–time integral during diastole; AP—augmentation pressure; AIx—augmentation index; AIx75—augmentation index normalized to a heart rate of 75 beats per minute; ESP—end-systolic pressure; HR duration—heart rate duration; ED—ejection duration; SEVR—subendocardial viability ratio; Pb—backward pressure wave; Pf—forward pressure wave; PPA—pulse pressure amplification; RM—reflection magnitude.

**Table 5:** Use of glycated hemoglobin percentage (HbA1c) for explaining parameters related to subclinical atherosclerosis and arterial stiffness. The model was adjusted for age, sex, body mass index (BMI), and smoking.

Parameter	β	95% CI	*p*
cIMT	0.103	−0.102; 0.307	0.318
log(cfPWV)	0.062	−0.177; 0.301	0.607
cSBP	−0.11	−0.388; 0.166	0.426
cPP	−0.025	−0.263; 0.213	0.837
cMAP	−0.182	−0.491; 0.128	0.244
MAP systole	−0.123	−0.413; 0.167	0.399
log(PTI systole)	−0.23	−0.537; 0.077	0.139
log(AP)	−0.201	−0.458; 0.056	0.123
AIx	−0.199	−0.474; 0.076	0.153
AIx75	−0.286	−0.566; −0.006	0.045
ESP	−0.167	−0.454; 0.121	0.25
Pb	−0.09	−0.332; 0.152	0.458
log(Pf)	0.009	−0.245; 0.264	0.942
log(PPA)	0.125	−0.15; 0.4	0.365
RM	−0.193	−0.472; 0.086	0.171

Abbreviations: cIMT—carotid intima–media thickness; cfPWV—carotid–femoral pulse wave velocity; cSBP—central systolic blood pressure; cPP—central pulse pressure; cMAP—central mean arterial pressure; MAP systole—mean arterial pressure during systole; PTI systole—pressure–time integral during systole; AP—augmentation pressure; AIx—augmentation index; AIx75—augmentation index normalized to a heart rate of 75 beats per minute; ESP—end-systolic pressure; Pb—backward pressure wave; Pf—forward pressure wave; PPA—pulse pressure amplification; RM—reflection magnitude.

## Data Availability

The data presented in this study are available on request from the corresponding author due to privacy restrictions.
